# Roles of proprotein convertase subtilisin/kexin type 9 (PCSK9) in the regulation of hepatic triglyceride metabolism

**DOI:** 10.1016/j.jlr.2026.101144

**Published:** 2026-09-10

**Authors:** Daniel Palenque-Blanco, Michele Alves-Bezerra

**Affiliations:** Department of Biomedicine, Biotechnology and Public Health, Biomedical Research and Innovation Institute of Cadiz (INiBICA), Faculty of Medicine, University of Cadiz, Cadiz, Spain

**Keywords:** PCSK9, lipid metabolism, lipoprotein metabolism, LDL, VLDL, CD36, MASLD, MASH

## Abstract

Proprotein convertase subtilisin/kexin type 9 (PCSK9) is a central regulator of cholesterol homeostasis, primarily by promoting the lysosomal degradation of the hepatic low-density lipoprotein receptor (LDLR). However, emerging clinical and preclinical evidence suggests that PCSK9’s influence extends beyond cholesterol clearance to the modulation of hepatic triglyceride metabolism. This review examines the biochemical mechanisms by which PCSK9 regulates intrahepatic triglyceride steady-state concentrations, highlighting its role in the assembly and lipidation of very low-density lipoproteins (VLDL) through both LDLR-dependent and LDLR-independent mechanisms. We further discuss the integration of PCSK9 activity with hepatic lipid-sensing mechanisms and de novo lipogenesis. Finally, we evaluate the impact of current PCSK9-targeted therapies on the hepatic lipidome and contextualize these findings within the pathogenesis of metabolic dysfunction-associated steatotic liver disease (MASLD). By highlighting the non-canonical roles of PCSK9, we aim to critically assess its potential as a multifaceted therapeutic target for dyslipidemias and steatotic liver diseases.

The liver is a central organ in the maintenance of systemic lipid and lipoprotein homeostasis, coordinating the uptake, synthesis, storage, oxidation, and export of major lipid classes such as fatty acids, triglycerides (TAG), and cholesterol. Lipids are essential not only as a major source of energy but also as structural components of cellular membranes and precursors for steroid hormones and signaling molecules. Because lipids are poorly soluble in plasma, their transport depends on lipoproteins, complex particles that mediate the distribution of triglycerides and cholesterol among tissues. In this context, the liver orchestrates the production and clearance of circulating lipoproteins, including very-low-density lipoproteins (VLDL) and high-density lipoproteins (HDL) while also regulating cholesterol disposal through bile acid synthesis and biliary secretion. Hepatic lipid metabolism is tightly controlled by nutritional, hormonal, and inflammatory signals, allowing metabolic adaptation to fasting and feeding states. Disruptions in this finely regulated network are linked to the pathogenesis of dyslipidemias, metabolic dysfunction-associated steatotic liver disease (MASLD), insulin resistance, and cardiovascular disease.

The liver is also the primary site of proprotein convertase subtilisin/kexin type 9 (PCSK9) synthesis and secretion (1). The canonical function of PCSK9 involves the regulation of systemic cholesterol homeostasis through the targeted endolysosomal proteolysis of hepatic low-density lipoprotein receptor (LDLR), which limits receptor recycling and impairs LDL-cholesterol clearance (2, 3). Beyond its canonical role in LDLR-mediated endocytosis, PCSK9 facilitates the production of VLDL through both LDLR-dependent and independent mechanisms (4, 5). Recent evidence also suggests that PCSK9 promotes the degradation of CD36, which may modulate fatty acid uptake into hepatocytes (6). Furthermore, PCSK9 has been implicated in hepatic inflammatory processes, potentially mitigating oxidative stress and cytotoxicity in the context of liver injury (7).

The discovery of PCSK9 had a transformative impact on clinical medicine, particularly in the prevention and management of atherosclerotic cardiovascular disease. Gain-of-function (GOF) mutations in *PCSK9* are associated with severe hypercholesterolemia and increased cardiovascular risk, whereas loss-of-function (LOF) variants confer lifelong reductions in circulating LDL-cholesterol levels and substantial protection against coronary artery disease. These observations established PCSK9 as a major therapeutic target and led to the rapid development of PCSK9-directed therapies, which have demonstrated remarkable efficacy in lowering LDL-cholesterol and reducing cardiovascular events. Beyond its established role in cardiovascular biology, the clinical relevance of PCSK9 is increasingly being recognized in the context of metabolic diseases, including MASLD, obesity, insulin resistance, and type 2 diabetes. Emerging evidence suggests that PCSK9 participates in multiple aspects of hepatic lipid handling, inflammatory signaling, and lipoprotein metabolism, thereby linking cholesterol homeostasis to systemic metabolic dysfunctions. Understanding how PCSK9 contributes to intrahepatic lipid accumulation and systemic metabolic alterations is therefore essential for refining current therapeutic approaches and for evaluating the potential metabolic consequences of long-term PCSK9 modulation.

In this review, we discuss the molecular regulation of PCSK9, its canonical and noncanonical functions in hepatic lipid metabolism, as well as its emerging role in the pathophysiology of metabolic liver diseases. We further compare evidence from preclinical models with available human genetic and clinical studies to critically evaluate how the modulation of PCSK9 contributes to intrahepatic lipid homeostasis and how these mechanisms relate to current liver-targeted therapeutic strategies.

## PCSK9 in Lipoprotein Metabolism

Seminal studies in cell and mouse models demonstrated that the primary regulatory function of PCSK9 is exerted through its interaction with the LDLR on the plasma membrane of hepatocytes. Synthesized as a ∼74 kDa precursor, PCSK9 undergoes intramolecular autocatalytic cleavage within the endoplasmic reticulum (ER), a maturation step that is strictly required for its subsequent secretion into the systemic circulation (8). Once secreted, PCSK9 facilitates the degradation of the LDLR through a non-enzymatic, pH-sensitive mechanism. The interaction is initiated when the catalytic domain of PCSK9 binds to the epidermal growth factor-like repeat A (EGF-A) domain of LDLR (9). Importantly, in this context PCSK9 functions primarily as a non-proteolytic scaffold rather than as an active protease, stabilizing an extended LDLR conformation involving the EGF-B domain that interferes with the conformational rearrangements required for receptor recycling and thereby redirects the receptor toward lysosomal degradation following endocytosis (10, 11).

Following binding, the PCSK9-LDLR complex is internalized via clathrin-mediated endocytosis (3, 10, 11). While the complex forms at a neutral physiological pH, its affinity increases approximately 150-fold within the acidic environment of the endosome (pH ∼5.5–6.0) (10). This prevents the LDLR from adopting the closed conformation required to release its LDL cargo and recycle back to the plasma membrane. Consequently, the complex is diverted to the lysosomal compartment, where it undergoes terminal proteolysis, reducing the surface density of functional LDLRs (3) (Fig. 1A). This targeted degradation impairs the liver's capacity for LDL-cholesterol (LDL-C) clearance, leading to elevated plasma levels and increased cardiovascular risk (3).

**Fig. 1:**
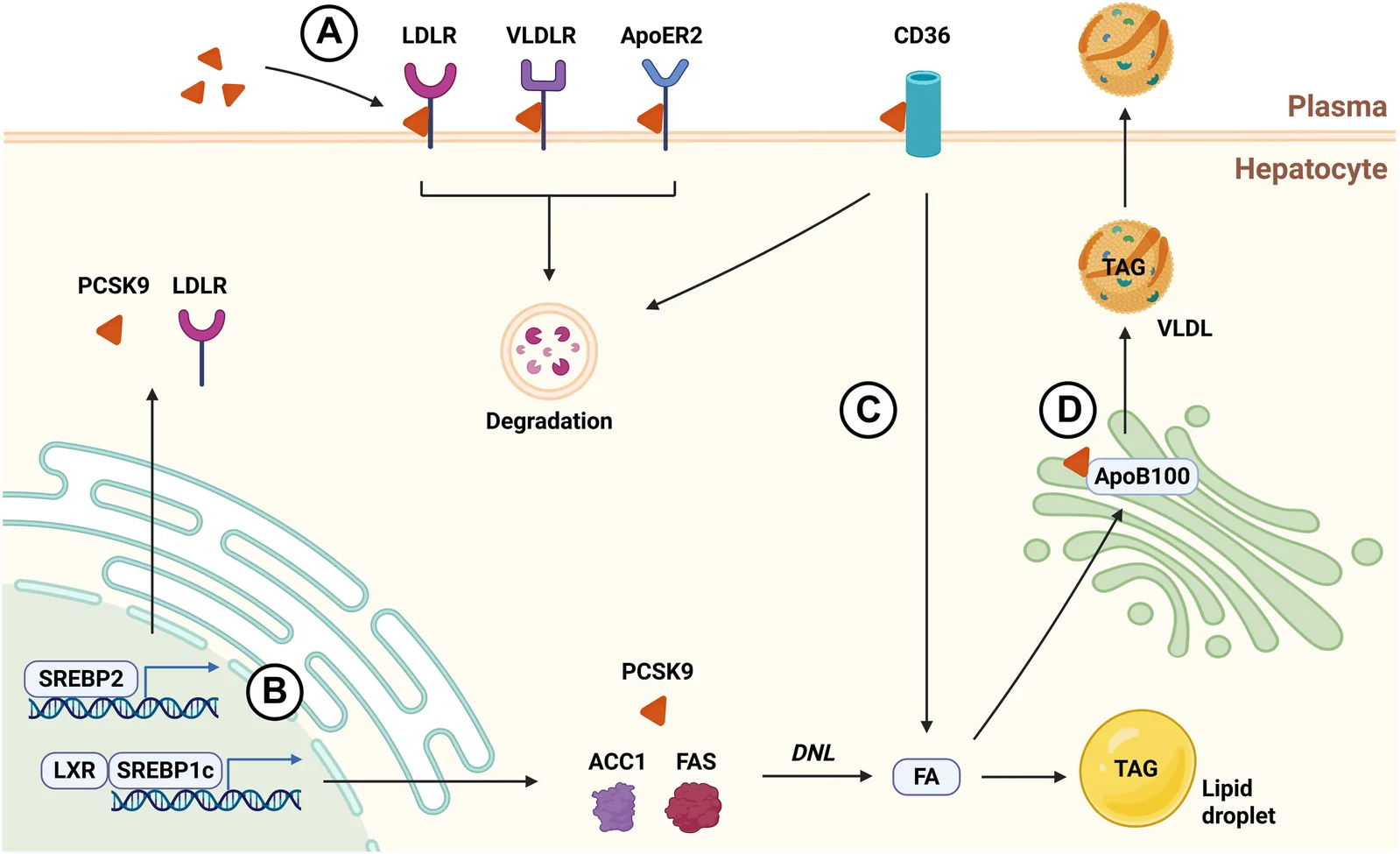
Molecular mechanisms by which PCSK9 regulates hepatic lipid metabolism. PCSK9 modulates hepatic lipid homeostasis through multiple interconnected pathways affecting lipoprotein metabolism, de novo lipogenesis, fatty acid uptake, and triglyceride secretion and storage. A: Extracellular PCSK9 promotes the clathrin-dependent endocytosis and lysosomal degradation of low-density lipoprotein receptor (LDLR). This impairs hepatic LDL-cholesterol (LDL-C) clearance, leading to elevated plasma levels. PCSK9 similarly targets the very-low-density lipoprotein receptor (VLDLR) and apolipoprotein E receptor 2 (ApoER2/LRP8) for degradation, reducing the clearance of triglyceride-rich lipoprotein (TRL) remnants and contributing to postprandial hypertriglyceridemia. B: Intracellular cholesterol depletion activates sterol regulatory element-binding protein 2 (SREBP-2), inducing the transcription of *PCSK9*/*Pcsk9* and *LDLR*/*Ldlr* in human and murine hepatocytes, thereby coupling cholesterol sensing to lipoprotein clearance. Concurrently, lipogenic stimuli activate the liver X receptor (LXR)/sterol regulatory element-binding protein 1c (SREBP-1c) pathway. This pathway upregulates *PCSK9*/*Pcsk9* expression alongside *Acaca* (encoding acetyl-CoA carboxylase 1, ACC1) and *FASN*/*Fasn* (encoding fatty acid synthase, FAS), robustly enhancing de novo lipogenesis (DNL). C: PCSK9 directly interacts with CD36, a fatty acid translocase on the plasma membrane, targeting it for lysosomal and proteasomal degradation. This mechanism presumably limits the uptake of extracellular long-chain fatty acids into the hepatocyte, independently of DNL-derived fatty acid pools. D: Intracellularly, PCSK9 stabilizes nascent apolipoprotein B100 (ApoB100), preventing its degradation, which facilitates very-low-density lipoprotein (VLDL) assembly and VLDL-triglyceride (VLDL-TAG) secretion into the plasma. Excess TAG is alternatively stored intracellularly within lipid droplets.

The clinical relevance of this pathway is underscored by genetic variants; LOF mutations (*e.g.*, Y142X) result in significantly lower LDL-C and lifelong cardiovascular protection (12). Furthermore, more prevalent LOF polymorphisms, such as R46L, have been directly linked to a reduction in subclinical vascular disease—demonstrated by a lower common carotid artery intima-media thickness—and a decreased risk of vascular-related conditions like erectile dysfunction, though these effects can be modulated by concurrent genetic factors such as ApoE alleles (13, 14). Conversely, GOF mutations (e.g., D374Y) exacerbate receptor degradation, leading to autosomal dominant hypercholesterolemia (15, 16).

Beyond its canonical role in LDLR regulation, studies in humans and preclinical models demonstrated that PCSK9 serves as a negative regulator of other LDLR family members sharing the conserved EGF-A domain, such as the VLDL receptor (VLDLR) and apolipoprotein E receptor 2 (ApoER2/LRP8) (4, 5, 17) (Fig. 1A). By promoting the degradation of these receptors, PCSK9 reduces the clearance of TAG-rich lipoprotein (TRL) remnants, contributing to postprandial hypertriglyceridemia. Furthermore, the influence of PCSK9 on ApoB-containing lipoproteins may involve distinct intracellular mechanisms. It has been postulated that PCSK9 acts as a chaperone-like stabilizer for nascent ApoB-100, protecting it from autophagic or proteasomal degradation and thereby facilitating the assembly and secretion of VLDL (5). While some studies suggest an influence on HDL metabolism, a direct degradative effect on receptors like SR-BI lacks robust primary evidence and is generally considered an indirect effect of broader systemic lipid remodeling (17).

Taken together, these roles position PCSK9 as a multifaceted regulator of lipoprotein metabolism, driving the lysosomal clearance of the LDLR, VLDLR, and ApoER2 while intracellularly safeguarding nascent ApoB-100 from degradation to facilitate VLDL assembly.

## PCSK9 and the hepatic lipid-sensing mechanisms

Human *PCSK9* and murine *Pcsk9* gene expression is directly regulated by core transcriptional pathways involved in hepatic lipid sensing, such as the Sterol Regulatory Element-Binding Protein (SREBP) family and the Liver X Receptor (LXR), which coordinate the liver's response to cholesterol and fatty acid availability.

The primary transcriptional activator of *Pcsk9* is SREBP-2, a master regulator of cholesterol homeostasis (2). When intrahepatic cholesterol levels are reduced, the SREBP cleavage-activating protein (SCAP)/SREBP complex moves from the ER to the Golgi. There, it is processed into its active nuclear form (nSREBP-2), which binds to sterol regulatory elements (SREs) in the promoter regions of target genes. This triggers the simultaneous transcription of murine *Pcsk9*, cholesterol synthesis enzymes (*e.g., Hmgcr*), and *Ldlr* (18, 19, 20), thereby tightly coupling cholesterol sensing to lipoprotein uptake and clearance (Fig. 1B). Further evidence linking PCSK9 to the cholesterogenic response was provided by seminal studies demonstrating the ability of statins and cholesterol depletion to enhance human *PCSK9* transcription (21). Thus, although the PCSK9 protein induces LDLR degradation, mRNA levels of both *PCSK9*/*LDLR* in humans and *Pcsk9*/*Ldlr* in rodents are positively regulated by cholesterol depletion and SREBP-2 activation. In addition, hepatocyte nuclear factor 1α (HNF1α) acts cooperatively with SREBP-2 to enhance *Pcsk9* promoter activity, contributing to the marked induction of PCSK9 protein observed during statin therapy in a dyslipidemic murine model (22).

Beyond cholesterol sensing, mouse *Pcsk9* is directly targeted and upregulated by the SREBP-1 isoforms, which are transcriptionally regulated downstream of LXR nuclear receptors and act as primary drivers of fatty acid synthesis (20). When SREBP transcriptional activity is induced—SREBP-2 in response to sterols or SREBP-1c under lipogenic stimuli—*Pcsk9* transcription is upregulated (Fig. 1B). The resulting PCSK9 protein directly interacts with CD36, a translocase involved in fatty acid transport across the plasma membrane, and targets it for lysosomal and proteasomal degradation (6, 20). Consequently, this PCSK9-mediated degradation of cell-surface CD36 presumably limits the uptake of extracellular long-chain fatty acids and the accumulation of triglycerides in the liver (6) (Fig. 1C). Conversely, the absence of PCSK9 leads to a significant increase in hepatic CD36 protein levels, increasing fatty acid uptake and lipid droplet accumulation, which enhances susceptibility to hepatic steatosis (6). Therefore, the induction of PCSK9/*Pcsk9* alongside SREBP-regulated de novo lipogenesis serves to restrict external fatty acid uptake when endogenous synthesis is active, preventing excessive hepatic triglyceride storage (6, 20).

These observations suggest that hepatic PCSK9 expression is tightly integrated into core sterol- and fatty acid-sensing networks via SREBP-2 and SREBP-1c, respectively. This mechanism likely establishes a dual-action feedback loop: alongside its canonical role in promoting LDLR degradation during cellular cholesterol depletion, PCSK9 simultaneously drives CD36 degradation to limit exogenous fatty acid uptake during periods of active endogenous lipogenesis, thereby safeguarding liver tissue from lipid-induced stress.

## Roles of PCSK9 in hepatic triglyceride metabolism

While the role of PCSK9 in cholesterol homeostasis is well defined in preclinical models and humans, its contribution to triglyceride metabolism involves an incompletely understood array of both LDLR-dependent and LDLR-independent pathways. Recent evidence suggests that PCSK9 acts as a significant modulator of hepatic triglyceride assembly, secretion, and storage.

### Impact of PCSK9 on ApoB-100-containing lipoproteins

The classical view of PCSK9 as exclusively a regulator of LDLR degradation has been revised by findings demonstrating that it also modulates the hepatic production of ApoB-containing lipoproteins through LDLR-independent mechanisms. While PCSK9 classically promotes LDLR degradation, preclinical models have shown that its overexpression increases circulating cholesterol even in the absence of the LDLR (23). Notably, PCSK9 enhances the secretion of TRLs and plasma levels of ApoB independent of the LDLR, as demonstrated in transgenic and LDLR-deficient mice (5).

ApoB synthesis is constitutive, but its intracellular stability is governed by lipid availability and microsomal triglyceride transfer protein (MTTP) activity (24). In mice, PCSK9 directly interacts with ApoB-100 at early stages of its processing, inhibiting its intracellular degradation via the autophagic pathway, thereby allowing more ApoB to escape degradation and be secreted (5) (Fig. 1D). This effect is accompanied by increased ApoB production at a post-transcriptional level, without changes in mRNA expression, suggesting enhanced protein stability or processing (5).Taken together, these mechanisms would contribute to increased plasma levels of ApoB, cholesterol, and triglycerides.

Additionally, in transgenic mice expressing human PCSK9 (hPCSK9), a substantial fraction of circulating hPCSK9 (approximately 25%–30%) is directly associated with plasma LDL, and elevated levels of plasma hPCSK9 positively correlate with increased TRL secretion, suggesting that a bidirectional relationship between PCSK9 and lipoprotein metabolism could be conserved in humans (23). However, direct evidence in humans remains limited. An in vivo kinetic study in two patients with autosomal dominant hypercholesterolemia harboring a *PCSK9* GOF mutation (S127R) demonstrated that plasma cholesterol dysregulation is primarily driven by the overproduction of ApoB-100-containing lipoproteins (VLDL, IDL, and LDL) (4). Although these clinical findings align with preclinical evidence, additional studies are needed to clarify the specific functions of PCSK9 in the production and secretion of ApoB-containing lipoproteins in human livers.

### Modulation of hepatic fatty acid uptake and de novo lipogenesis

Beyond ApoB stabilization, PCSK9 may impact hepatic lipid flux by regulating CD36. In mice, PCSK9 promotes the degradation of CD36 through both lysosomal and proteasome-sensitive pathways (6). This effect is supported by experimental evidence demonstrating that proteasomal inhibition with MG132 attenuates PCSK9-mediated CD36 degradation. The interaction between PCSK9 and CD36 leads to reduced translocase levels at the cell surface, likely limiting fatty acid uptake (6) (Fig. 1C). This regulatory mechanism is selective, as PCSK9 does not appear to affect other related receptors such as SR-BI or LIMP2 (6). Nevertheless, interpreting the overall impact of PCSK9 on hepatic fatty acid uptake requires accounting for the cell type-specific expression and complex functionality of CD36 in the liver. Under basal physiological conditions, CD36 expression is relatively low in hepatocytes compared with non-parenchymal cells, such as Kupffer cells. However, CD36 mRNA and protein expression are markedly induced in humans and mice under metabolic stress—such as diet-induced obesity and MASLD—correlating with fatty acid influx and intrahepatic triglyceride storage (25, 26). Whether PCSK9 primarily modulates baseline fatty acid handling in non-parenchymal cells or serves as an adaptive mechanism against hepatocyte lipotoxicity during metabolic stress remains an important open question.

In addition to its putative role in fatty acid uptake, PCSK9 has been linked to the regulation of de novo lipogenesis (DNL). In transgenic mice, hPCSK9 expression promotes hepatic DNL through the upregulation of *Srebf1* (encoding SREBP-1c) and its downstream lipogenic targets, *Acaca* (encoding acetyl-CoA carboxylase 1, ACC1) and *Fasn* (encoding fatty acid synthase, FAS) (27) (Fig. 1B). Notably, PCSK9 regulates these pathways at both transcriptional and post-transcriptional levels. Under standard dietary conditions, hPCSK9 expression increases hepatic FAS protein abundance by approximately 2-fold without altering *Fasn* mRNA expression, whereas under a high-fat diet, this effect is markedly enhanced, resulting in a sixfold increase in FAS protein levels (27). Importantly, the induction of lipogenic enzymes by hPCSK9 is strictly dependent on the presence of both LDLR and Apolipoprotein E (ApoE), as genetic deletion of either component completely abolishes activation of SREBP-dependent lipogenic pathways (27).

Whether these lipogenic mechanisms operate to the same extent in humans remains unclear. Clinical studies in patients with MASLD demonstrated that both circulating PCSK9 concentrations and hepatic *PCSK9* mRNA expression are positively associated with the severity of steatosis and with increased hepatic expression of SREBP-1c and FAS (28). In a separate cohort of morbidly obese patients with MASLD, hepatic *PCSK9* mRNA levels were positively correlated with *FASN*, *PPARG* (encoding PPARγ), and *PPARA* (encoding PPARα) (29). Nonetheless, while circulating PCSK9 levels were associated with the severity of hepatic steatosis, no significant correlation was observed between hepatic *PCSK9* mRNA expression and the degree of hepatic steatosis in this study (29).

Collectively, these mechanisms likely contribute to elevated hepatic fatty acid availability, leading to increased intrahepatic triglyceride storage (30). However, direct evidence linking intrahepatic PCSK9 to hepatic lipid synthesis in humans is still scarce, underscoring the need for further translational studies.

### Intracellular triglyceride storage and secretion

Given the direct and indirect mechanisms by which PCSK9 modulates hepatic lipid homeostasis, changes in PCSK9 levels are expected to affect the balance between lipid droplet storage, lipolytic mobilization, and VLDL assembly.

Under basal conditions, intrahepatic triglycerides are dynamically stored within cytosolic lipid droplets and may undergo lipolysis—primarily mediated by adipose triglyceride lipase (ATGL) and hormone-sensitive lipase (HSL)—to supply fatty acids for oxidation or re-esterification. By stabilizing nascent ApoB-100 and preventing its autophagic degradation, PCSK9 presumably favors the trafficking of newly synthesized triglycerides toward the VLDL secretory pathway rather than allowing their storage into lipid droplets (5) (Fig. 1D). In this context, PCSK9 would promote VLDL-TAG packaging to the detriment of lipid droplet storage.

However, in conditions where VLDL secretion capacity is saturated, the excess fatty acids generated by PCSK9-mediated DNL may be diverted back into cytosolic storage, promoting hepatocyte steatosis (30). This intrahepatic accumulation would be further aggravated at the systemic level by the loss of surface LDLRs, which severely hinders the receptor-mediated re-uptake and clearance of circulating VLDL and intermediate-density lipoprotein (IDL) remnants (31).

Thus, by simultaneously modulating DNL, ApoB stability, and receptor-mediated clearance, PCSK9 presumably serves as a key regulator that determines whether hepatic lipids are channeled into intracellular storage or secretion (Fig. 1).

## PCSK9 in the Pathogenesis of Steatotic Liver Diseases

### Circulating PCSK9 and MASLD progression

In clinical cohorts, circulating PCSK9 levels correlate with the severity of hepatic fat accumulation and secondary tissue damage. Elevated plasma PCSK9 concentrations are positively associated with the histological grades of hepatic steatosis, necroinflammation, and tissue fibrosis (28). This association remains robust when steatosis is quantified using non-invasive imaging and fatty liver indices across large-scale cohorts (32).

To explain this clinical correlation, two distinct pathophysiological paradigms have been proposed. First, elevated PCSK9 may actively drive MASLD development by chronically upregulating hepatic DNL in response to altered intracellular cholesterol pools (27). Alternatively, it may act indirectly through a compensatory mechanism whereby hepatic fat accumulation induces increased PCSK9 production in an attempt to limit cholesterol uptake by already overloaded hepatocytes (19, 28).

PCSK9 appears to play a causal role in hepatic steatosis and metabolic dysfunction-associated steatohepatitis (MASH). In experimental MASH models, hepatic and circulating PCSK9 levels are markedly increased, whereas genetic deletion or liver-specific silencing of PCSK9 attenuates hepatic steatosis, inflammation, and fibrosis. These protective effects are associated with reduced activation of the TLR4/NF-kB pathway, lower expression of pro-inflammatory cytokines, and decreased fibrogenic markers, supporting a direct role for PCSK9 in the progression from steatosis to steatohepatitis and fibrotic liver injury (33). Interestingly, the pathological role of PCSK9 may not be restricted to metabolic liver disease. In experimental models of alcohol-associated liver disease (ALD), PCSK9 inhibition reduces hepatic steatosis, inflammation and oxidative stress, suggesting that PCSK9 may represent a common mediator of chronic liver damage across distinct etiologies (34).

Human genetic studies further provide evidence supporting a direct, causative role for PCSK9 in modulating tissue vulnerability to steatosis and subsequent fibrosis. Specific rare missense mutations, such as the p.Gly316Cys variant, have been linked to an increased susceptibility to mild-to-moderate steatosis without accelerating advanced fibrosis (35). Conversely, carrying the well-characterized LOF variant rs11591147 (p.R46L) provides significant clinical protection against the full spectrum of MASLD progression. In a large-scale European biopsy-controlled cohort, carriers of this LOF allele exhibited a markedly lower risk of developing hepatic steatosis, steatohepatitis, and severe liver fibrosis (36). This protective genetic effect operates independently of classic metabolic risk factors, as well as established risk alleles in *PNPLA3* and *TM6SF2*, demonstrating that variations in endogenous PCSK9 activity fundamentally alter the trajectory of chronic liver injury.

### PCSK9 confers protection against lipotoxicity

The modulation of hepatic lipid handling by PCSK9 extends to the regulation of inflammatory responses and lipotoxicity. Experimental evidence in both animal models and hepatoma cell lines demonstrates that PCSK9 deficiency accelerates hepatic lipid uptake and accumulation, a process potentially driven by the post-translational upregulation of the scavenger receptor CD36. Beyond its role in lipid trafficking, physiological expression of PCSK9 exerts a cytoprotective effect, shielding hepatocytes from fatty acid-induced ER stress and cytotoxicity—pathways that are central to the development of hepatic steatosis and liver injury.

In vitro studies in HepG2 cells reveal that PCSK9 deficiency exacerbates cellular susceptibility to palmitate-induced lipotoxicity, resulting in the marked accumulation of reactive oxygen species (ROS) and enhanced oxidative stress. Conversely, the presence of functional PCSK9 mitigates these lipotoxic effects, demonstrating a protective role against lipid-induced oxidative damage (7).

Mechanistically, extracellular or autocrine PCSK9 may mediate this cytoprotection through its direct interaction with CD36. By facilitating the degradation of CD36, native PCSK9 is expected to restrict exogenous fatty acid influx, thereby limiting lipotoxicity. In the absence of PCSK9, the unchecked upregulation of cell-surface CD36 drives an increase in the influx of fatty acids into mouse livers, triggering sustained ER stress and cytotoxicity (6). Consequently, under conditions of acute metabolic stress, physiological levels of PCSK9 may function to restrict lipid-induced oxidative damage and liver injury.

While animal models and in vitro systems clearly highlight a cytoprotective role for PCSK9 against lipid overload and oxidative stress, clinical evidence in humans remains remarkably scarce. Given known species-specific variations in hepatic lipid handling and PCSK9 regulation, further clinical studies are needed to determine whether these protective mechanisms translate to human metabolic liver disease.

### Preclinical and clinical evidence: Consequences of PCSK9 loss- and gain-of-function mutations

The roles of PCSK9 in regulating systemic lipoprotein clearance and intrahepatic lipid handling are supported by distinct metabolic phenotypes resulting from targeted gene knockout, retention mutations, or naturally occurring variants under dietary stress.

In global germline knockout (*Pcsk9*^−/−^) models, high-fat diet (HFD) feeding induces severe hepatic steatosis and accelerates liver injury (7). Mechanistically, the complete absence of PCSK9 disrupts the SREBP2-PCSK9-LDLR and CD36 regulatory axes, preventing the liver from downregulating surface LDLR in response to the lipid overload. This forces a persistent, receptor-mediated internal trapping of circulating lipoproteins within an already lipid-overloaded tissue (7). Concurrently, the lack of intracellular PCSK9 impairs nascent ApoB-100 stability and limits VLDL core assembly, resulting in an export defect. Consequently, total ablation of PCSK9 leads to metabolic reprogramming towards exacerbated intrahepatic lipid storage, resulting in increased lipotoxicity, liver injury, and MASH (7).

A distinct hepatic phenotype is observed with the human *PCSK9*^Q152H^ variant, an uncleavable LOF mutation. This mutant protein cannot undergo the intra-ER autocatalytic cleavage required for secretion. The retention of the proPCSK9 variant within the ER does not cause cellular injury; rather, it triggers a localized, protective adaptive response. Hepatocytes harboring the *PCSK9*^Q152H^ mutation exhibit a marked upregulation of luminal ER chaperones, specifically glucose-regulated protein 78 (GRP78) and GRP94. This coordinated chaperone response preserves protein-folding capacity during overnutrition, shielding the liver tissue from fatty acid-induced ER stress, apoptosis, and subsequent diet-induced liver injury (37).

This protective profile driven by PCSK9 deficiency aligns with clinical genetic data evaluating naturally occurring human LOF variants, such as rs11591147 (R46L). Epidemiological cohorts demonstrate that carriers of the R46L LOF allele exhibit reduced circulating LDL cholesterol levels and are protected against the development of MASLD/MASH (36). Conversely, GOF PCSK9 variants drive severe hypercholesterolemia by decreasing hepatic LDLR availability (38, 39), with specific variants like S127R linked to the overproduction of ApoB-100-containing lipoproteins (VLDL, IDL, and LDL) (4). Nevertheless, the specific impact of PCSK9 GOF variants on metabolically-induced liver injury and MASLD remains to be determined clinically.

Collectively, these observations demonstrate that the metabolic outcomes of PCSK9 dysregulation are dictated by multiple factors such as the underlying molecular mechanism, tissue context, and gene dosage. While complete germline ablation in rodents forces intrahepatic lipid trapping and accelerates liver injury under overnutrition, human LOF phenotypes—whether driven by adaptive ER retention or reduced circulating activity—protect against lipotoxicity and MASLD/MASH. Reconciling these divergent phenotypic profiles and resolving the uncharacterized hepatic effects of GOF variants will require refined preclinical models and extensive clinical data. Furthermore, addressing the male-cohort bias in preclinical studies remains essential to evaluate how sex-based dimorphisms influence PCSK9-mediated hepatic lipid homeostasis.

## Therapeutic Implications: Beyond LDL-C lowering

The clinical success of PCSK9 inhibitors has brought to light important considerations regarding systemic lipid clearance and its impact on intrahepatic lipid homeostasis (40). By preventing the non-enzymatic, endolysosomal degradation of LDLR, pharmacological inhibition of PCSK9 preserves high surface receptor densities on hepatocytes (41), accelerating the internalization LDL and ApoE-containing remnant particles. Early metabolic models hypothesized that this persistent lipid influx could overwhelm hepatocellular processing pathways, thereby predisposing the liver to steatosis. However, multi-center clinical trials and long-term cohort evaluations demonstrate that the human liver adapts to this increased lipoprotein burden without establishing or exacerbating hepatic steatosis. Clinical evidence indicate that therapeutic inhibition of circulating PCSK9 effectively reduces systemic atherosclerotic cardiovascular risk while preserving intrahepatic metabolic balance, a profile that extends safely to patients presenting with pre-existing MASLD and type 2 diabetes (42).

To understand how hepatocytes avoid lipotoxicity under these conditions, it is essential to examine the profound lipidomic changes induced by PCSK9 modulation. Complete deficiency or targeted inhibition of PCSK9 significantly remodels the hepatic lipidome, preventing the accumulation of pathogenic, saturated lipid species (42, 43). In mouse models, PCSK9 deficiency shifts the hepatic lipid profile toward a higher proportion of monounsaturated triglycerides and ceramides while reducing saturated lipid species, which are known to exert pro-inflammatory, pro-apoptotic and lipotoxic effects in the liver (43). This protective lipidomic shifting is closely tied to improvements in peripheral and hepatic insulin sensitivity, as well as a reduction in ectopic lipid deposition within MASLD models (43, 44). Furthermore, in patients with MASLD and type 2 diabetes PCSK9 inhibition has been associated with reductions in circulating sphingolipids, including ceramides and related metabolites, reinforcing its potential role in attenuating lipid-induced hepatic injury and metabolic dysfunction (42).

While hepatic PCSK9 expression undergoes tight transcriptional regulation by nutritional status via insulin-driven SREBP-1c pathways (19), its direct inhibition induces key compensatory changes in several lipid metabolic pathways. These metabolic adaptations protect hepatocytes from lipid-induced ER stress and subsequent apoptotic cascades. Most notably, while the loss of active PCSK9 can upregulate cell-surface CD36 expression, clinical safety is preserved because therapeutic PCSK9 inhibition simultaneously lowers circulating TRL and triggers a favorable lipidomic remodeling that prevents the accumulation of lipotoxic saturated fatty acids (6).

Consequently, these pathways expand the therapeutic relevance of PCSK9 targeting, demonstrating its potential to improve hepatic lipid handling and modulate tissue metabolism beyond the simple control of circulating LDL-C levels (45). In line with that, pharmacological inhibition of PCSK9 achieves robust cardiovascular protection without inducing hepatic steatosis, driving a profound intrahepatic lipidomic shift away from lipotoxic, saturated species toward protective monounsaturated lipid profile.

## Conclusion and outlook

The clinical evolution of PCSK9 therapeutics underscores a key transition from systemic lipid reduction to a more nuanced appreciation of tissue-specific metabolic remodeling. Humanized monoclonal antibodies and liver-targeted siRNAs achieve comparable, robust reductions in major adverse cardiovascular events by maximizing LDLR recycling (40). However, because monoclonal antibodies neutralize extracellular pools while siRNAs arrest intracellular synthesis, these modalities trigger distinct intracellular feedback responses and homeostatic adaptations within both hepatic and extrahepatic tissues (45).

To date, inclisiran represents the most clinically advanced liver-targeted PCSK9-silencing therapy and therefore provides a unique opportunity to evaluate the metabolic consequences of reducing intrahepatic PCSK9 expression in humans. While inclisiran consistently produces robust reductions in LDL cholesterol, its effects on plasma triglycerides are comparatively modest. These findings suggest that, despite the marked effects observed in experimental models, intrahepatic PCSK9 may have a more limited contribution to hepatic lipogenesis and VLDL secretion in humans (46).

While extensive patient data confirm the systemic safety and efficacy of these interventions on standard lipid panels, translating these findings into a complete model of intrahepatic TRL dynamics leaves several critical areas unresolved. Specifically, while extracellular clearance is well-mapped, the exact intracellular mechanisms by which endogenous PCSK9 acts as a chaperone to stabilize nascent ApoB-100 remain poorly defined under conditions of therapeutic interventions and variable nutritional and metabolic states. Furthermore, despite abundant clinical data tracking circulating lipids, we still lack clarity on how these therapies alter the cell-surface stability of related receptors sharing the conserved EGF-A binding domain, such as VLDLR and ApoER2. Resolving this cross-regulation is essential to fully explain individual variability in postprandial remnant clearance among patient cohorts.

Finally, although preclinical models warn of altered intrahepatic lipid partitioning under absolute PCSK9 deficiency, clinical safety endpoints in humans remain heavily reliant on non-invasive serum biomarkers and imaging surrogates. Longitudinal, multi-center studies integrating human genetics, liver imaging, metabolic phenotyping, and paired tissue biopsies are therefore necessary to definitively map long-term liver safety, steatosis resolution, and fibrotic outcomes. This tissue-level validation represents an absolute prerequisite as the therapeutic paradigm shifts from transient, reversible inhibition of PCSK9 toward permanent genomic modifications, such as CRISPR/Cas9 base editing. Ultimately, mapping the precise tissue-specific, intracellular networks of PCSK9 will be essential to guarantee long-term hepatic safety alongside robust systemic lipid control.

## Data availability

All data supporting this study are included in the manuscript.

## Declaration of Generative AI and AI-Assisted Technologies in the Writing Process

During the preparation of this work, the authors used an AI-assisted tool (Gemini, Google) for language editing. All scientific content, interpretations, and conclusions were developed and verified by the authors. The authors take full responsibility for the content of this article.

## Conflict of interest

The authors declare that they have no conflicts of interest with the contents of this article.
